# Sepsis-associated disseminated intravascular coagulation and its differential diagnoses

**DOI:** 10.1186/s40560-019-0387-z

**Published:** 2019-05-20

**Authors:** Toshiaki Iba, Eizo Watanabe, Yutaka Umemura, Takeshi Wada, Kei Hayashida, Shigeki Kushimoto, Japanese Surviving Sepsis Campaign Guideline Working Group for disseminated intravascular coagulation, Hideo Wada

**Affiliations:** 1 Department of Emergency and Disaster Medicine, Juntendo University Graduate School of Medicine, 2-1-1 Hongo Bunkyo-ku, Tokyo, 113-8421 Japan; 2 Department of General Medical Science Graduate School of Medicine Chiba University, Chiba, Japan; 3 Department of Emergency and Critical Care Medicine Eastern Chiba Medical Center, Chiba, Japan; 4 Department of Traumatology and Acute Critical Medicine, Osaka University Graduate School of Medicine, Osaka, Japan; 5 Division of Acute and Critical Care Medicine, Department of Anesthesiology and Critical Care Medicine, Hokkaido University Graduate School of Medicine, Sapporo, Japan; 6 Department of Emergency and Critical Care Medicine, School of Medicine, Keio University, Tokyo, Japan; 7 Division of Emergency and Critical Care Medicine, Tohoku University Graduate School of Medicine, Sendai, Japan; 8 Department of Molecular and Laboratory Medicine, Mie University School of Medicine, Tsu, Japan

**Keywords:** Disseminated intravascular coagulation, Sepsis, Thrombotic thrombocytopenic purpura, Hemolytic uremic syndrome, Heparin-induced thrombocytopenia

## Abstract

Disseminated intravascular coagulation (DIC) is a common complication in sepsis. Since DIC not only promotes organ dysfunction but also is a strong prognostic factor, its diagnosis at the earliest possible timing is important. Thrombocytopenia is often present in patients with DIC but can also occur in a number of other critical conditions. Of note, many of the rare thrombocytopenic diseases require prompt diagnoses and specific treatments. To differentiate these diseases correctly, the phenotypic expressions must be considered and the different disease pathophysiologies must be understood. There are three major players in the background characteristics of thrombocytopenia: platelets, the coagulation system, and vascular endothelial cells. For example, the activation of coagulation is at the core of the pathogenesis of sepsis-associated DIC, while platelet aggregation is the essential mechanism in thrombotic thrombocytopenic purpura and endothelial damage is the hallmark of hemolytic uremic syndrome. Though each of the three players is important in all thrombocytopenic diseases, one of the three dominant players typically establishes the individual features of each disease. In this review, we introduce the pathogeneses, symptoms, diagnostic measures, and recent therapeutic advances for the major diseases that should be immediately differentiated from DIC in sepsis.

## Background

Sepsis is currently defined as “dysregulated host immune response to infection leading to organ failure” [1], and the severity of organ dysfunction has been recognized as a significant prognostic factor. The degree of organ dysfunction is usually evaluated using the Sequential Organ Failure Assessment (SOFA) score [2], and hematological failure is represented by the platelet count when determining the SOFA score. Thus, thrombocytopenia is often a clue to the presence of disseminated intravascular coagulation (DIC), and a platelet count below 50 × 109 L^−1^ is reported to be strongly associated with a poor outcome in patients with sepsis [3]. Platelet depletion is thought to arise from platelet consumption in the coagulopathy that occurs during sepsis, but platelets also aggregate actively to prevent bacterial dissemination [4, 5]. This means that platelets and the coagulation system cooperate together to enable host defense [6]. Indeed, the prothrombotic reaction elicited by platelet aggregation, activated coagulation, and endothelial damage is purposive**,** but it also is a preceding risk of sepsis [7]. Thus, the active application of anticoagulants for sepsis-associated DIC is recommended in some countries [8]. Although the platelet count is routinely measured in critical care settings, rare diseases that should be discriminated from sepsis-associated DIC have not received sufficient attention. Since many of these conditions require urgent treatment, clinicians must have sufficient knowledge of rare thrombocytopenic disorders. The International Society for Thrombosis and Hemostasis (ISTH) released guidelines for the differentiation of sepsis-associated DIC from other thrombocytopenic conditions in 2018 [9]. In conjunction with those guidelines, the Working Group for DIC of the Japanese Surviving Sepsis Campaign Guideline 2020 has highlighted the most important thrombocytopenic diseases. In addition, practical steps for differentiating these thrombocytopenic diseases are proposed in the present review.

Other than the above, we also wish to emphasize the importance of fully understanding the pathophysiology of thrombocytopenia. There are three major responsible players involved in thrombocytopenia: platelets, the coagulation system, and the vascular endothelium. Recognition of the individual mechanism of platelet depletion can help to classify thrombocytopenic diseases and to explain the differences in their clinical features [10]. For example, kidney injury is common in both hemolytic uremic syndrome (HUS) and thrombotic thrombocytopenic purpura (TTP); however, kidney injury is predominant and more intense in HUS, with incidences as high as 58% in TTP and 100% in HUS. Approximately 30% to 40% of patients with renal failure and HUS require renal support [10, 11], whereas severe renal failure and chronic renal insufficiency are rare in TTP. These differences in the aforementioned clinical courses can be explained by the individual disease pathogeneses: kidney injury in TTP is primarily caused by platelet aggregation in the vasculature, whereas kidney injury in HUS arises from damage to the glomerular endothelial cells and the resident renal cells [12]. In this review, we will also discuss the pathophysiology of various thrombocytopenic conditions.

## Main text

### Sepsis-associated disseminated intravascular coagulation

DIC is a common complication in sepsis, and its diagnosis is typically made based on some combination of basic coagulation markers [13, 14]. As an inevitable result, the specificity of such diagnoses is not sufficiently high. Thus, the ability to discriminate DIC from other thrombocytopenic diseases is essential [15]. Unfortunately, most patients with thrombocytopenia arising from other causes are initially diagnosed as having DIC, and the opportunity to treat such patients correctly can be missed. Sepsis-associated DIC is characterized by the systemic activation of coagulation and organ dysfunction complications arising from a microcirculatory disorder [16, 17]. The main laboratory features are thrombocytopenia, elevated levels of fibrin-related markers, and the consumption of coagulation factors. A hallmark of DIC is the combination of platelet depletion, increased fibrinogen/fibrin degradation products, and a prolonged prothrombin time (PT). These changes represent the microcirculatory disorder and, consequently, reflect the severity of sepsis [13, 18].

Coagulation plays an important role in the innate immune system and is closely linked to other inflammatory responses [6, 19]. The concept of “immunothrombosis” describes the interaction between coagulation and innate immunity [20]. Classically, the activation of coagulation is considered to be triggered by the expression of tissue factor on monocytes and macrophages by microorganisms and their components, described as pathogen-associated molecular patterns (PAMPs) [21]. Tissue factor is known to be a strong initiator of coagulation [22], but it also induces proinflammatory responses via the activation of protease-activated receptors (PARs) [22], but it also induces proinflammatory responses via the activation of PARs [23]. More recently, phosphatidylserine on the cellular membrane has been identified as another important coagulation activator [24]. In addition, other than PAMPs, damage-associated molecular patterns (DAMPs) released from damaged cells, which include structures such as cell free-DNA, histones, and high-mobility group box 1 protein (HMGB1), have been reported to participate in the activation of coagulation [6]. Similarly, neutrophil extracellular traps (NETs) comprised of mesh-like DNA fibers, nuclear proteins, and antimicrobial peptides have also been shown to accelerate thrombogenicity [6]. In addition to the activation of coagulation, another important feature of sepsis-associated DIC is the suppression of fibrinolysis. Plasminogen activator inhibitor-1 (PAI-1) released from damaged endothelial cells suppresses fibrinolysis, allowing the typical thrombotic phenotype of coagulopathy to appear [25].

### Thrombotic microangiopathy

#### Thrombotic thrombocytopenic purpura

Thrombotic microangiopathy (TMA) is characterized by massive thrombus formation in microvessels that leads to thrombocytopenia and microangiopathic hemolytic anemia (MAHA); in severe cases, it can lead to organ injuries [26]. TMA includes thrombotic thrombocytopenic purpura (TTP), HUS, and secondary TMA arising from various backgrounds. Both DIC and TTP cause microvascular thrombosis, but the thrombosis occurs mainly in postcapillary venules in DIC, while it occurs mainly in arterioles in TMA.

Acquired TTP is an autoimmune disease caused by the autoantibody-induced depletion or inhibition of a disintegrin and metalloproteinase with a thrombospondin type 1 motif, member 13 (ADAMTS13). TTP is often triggered by infection, and its discrimination from sepsis-associated DIC is not necessarily easy in such cases [27]. In TTP, microthrombi are induced by platelet/von Willebrand factor (VWF) microaggregate formation, and severe platelet depletion is the major characteristic. Common features of TTP consist of the pentad of thrombocytopenia, MAHA, fluctuating neurological signs, renal impairment, and fever. Since the incidence is far less than that of DIC [28], TTP is often initially diagnosed as DIC in sepsis patients. However, the prompt differentiation of TTP from DIC and the early initiation of plasma exchange is extremely important for successful treatment. The ADAMTS13 level in patients with TTP is commonly reduced to below 10%, and measurement of this parameter is the most reliable test for a definitive diagnosis [27]. Although a rapid ADAMTS13 activity assay with a turnaround time of several hours has been proposed recently [29], its use has not yet become prevalent. The ADAMTS13 level is also known to decrease in sepsis, but the level is usually maintained at above 30% [30]. In addition to the reduction in ADAMTS13, anti-ADAMTS13 antibodies and ultra-large VWF multimers can be detected in TTP patients who are in remission [31, 32]. The types of organ injuries differ slightly between DIC and TTP. The lung and cardiovascular system are common target organs in sepsis-associated DIC [33], while the kidney and brain are more common in TMA [34]; this difference is thought to be due to differences in the target vessels. Fibrin thrombi are commonly seen in the postcapillary venules in acute lung injury and septic shock [35]. In contrast, thrombi mainly formed by activated platelets were often predominantly seen in arteriole in the kidney and brain in the patients with TMA [12, 28]. Regarding treatment, once TTP is suspected, British guidelines recommend the initiation of plasma exchange within 4–8 h, and subsequent confirmation of a decreased ADAMTS13 level after the start of treatment is permitted [27]. Acute TTP is almost universally fatal without adequate treatment, but the mortality rate can be reduced to below 20% with the prompt delivery of plasma exchange. A novel treatment using caplacizumab, an anti-VWF A1 antibody, was recently approved in Europe for the treatment of acquired TTP, to be used in conjunction with plasma exchange and immunosuppression [36, 37]. Currently, an ADAMTS13 factor concentrate is being examined in a clinical trial, and the efficacy of recombinant ADAMS13 has been reported [38].

#### Shiga toxin-producing *Escherichia coli*-HUS

Shiga toxin-producing *Escherichia coli* (STEC) provokes severe hemorrhagic colitis, MAHA, and renal failure mainly in children, and needs to be differentiated from sepsis-associated DIC. The most severe cases of Shiga toxin-producing *Escherichia coli*-HUS (STEC-HUS) result in toxic megacolon and transmural necrosis of the colon with perforation, and the mortality rate remains high for such cases [39]. STEC-HUS has become rare in developed countries but is still common in developing countries [40]. Generally, the pathogenetic mechanism driving STEC-HUS is gastrointestinal infection with specific types of *E*. *coli*, typically O157: H7, O104: H4, or others [40, 41]. In this type of HUS, the toxin triggers the deposition of endothelial complement via endothelial injury and possibly interferes with the activity of complement regulatory molecules. It is also indicated that Shiga-toxin can stimulate VWF secretion from endothelial cells and this phenomenon may play an important role [42]. Like other TMAs, STEC-HUS is characterized by MAHA; therefore, RBC fragmentation (described as “schistocytes”) occurring in association with thrombus formation in arterioles is commonly seen in blood film. In contrast, RBC fragmentation is less common in DIC, since the dominant site of thrombus formation is in microvenules. Like other TMAs, dysfunction of the affected organs, specifically the kidneys and central nervous system, is the typical feature [43]. Culture-based assays, serological assessment, and polymerase chain reaction using a stool or rectal swab are useful for the detection of STEC. Once a patient is diagnosed as having STEC-HUS, appropriate fluid and electrolyte management, the avoidance of antidiarrheal drugs, and possibly the avoidance of antibiotic therapy are recommended as best practices [44]. Since the vicious cycle of complement activation, endothelial cell damage, platelet activation, and fibrin deposition is common with atypical HUS (aHUS) [43], complement inhibition might be the treatment of choice [42]. However, the effectiveness of anti-C5 monoclonal antibody (eculizumab) for the treatment of STEC-HUS has not been confirmed [44, 45]. Though this type of HUS is termed STEC-HUS, *E*. *coli* is not the only pathogen; patients with pneumococcal HUS are known to have a severe clinical picture, with MAHA, respiratory distress, and neurological involvement [46]. *Streptococcus pneumoniae hemolytic* is also known to causes HUS in children [47].

#### Atypical HUS

aHUS is a rare disease that is often recognized by the presence of thrombocytopenia, MAHA, acute kidney injury, and other organ dysfunctions. Since aHUS-specific therapeutics have now been introduced, prompt discrimination from sepsis-associated DIC is mandatory. However, the similarity in clinical presentations and the absence of a definitive diagnostic test have hindered the selection of a therapeutic target [48]. aHUS results from the uncontrolled activity of the alternative complement pathway and is often triggered by infection, which activates platelets, induces hemolysis, and damages the vascular endothelium. The laboratory findings of aHUS are represented by thrombocytopenia (< 150 × 10^9^ L^−1^), hemolytic anemia (RBC fragmentation, elevated lactate dehydrogenase [LDH], elevated bilirubin, decreased hemoglobin [< 10 g/dL], and depleted haptoglobin), and organ failure [43]. Though an evaluation of the complement system is helpful for diagnosis, only two-thirds of aHUS cases are associated with an identifiable complement-activating condition, and no abnormalities are detectable in the rest. Complement factor testing for complement component 3 and complement component 4, complement factor H, complement factor I, and antibodies against complement components can help to detect protein deficiencies [49]. Concurrently, genetic testing is sometimes, but not always, useful for the immediate diagnosis of aHUS. Genetic abnormalities are reportedly found in approximately 50% to 70% of patients with aHUS [50]. Among them, mutations in complement factor H account for approximately 25% of aHUS cases, membrane cofactor protein for approximately 10%, complement factor I for 5% to 10%, and thrombomodulin for up to 5% [51]. Multiple *C3* mutations have been detected in 5–10% and 31% of aHUS cases in Caucasian and Japanese populations, respectively [52]. There is no clinically available specific diagnostic tool for aHUS; therefore, obtaining a complete patient medical and family history is extremely important. Notably, an atypical clinical course for sepsis-induced DIC, such as sustained symptoms after the resolution of infection, often becomes a clue for the diagnosis of aHUS. In summary, since there is no reliable definitive test, aHUS is usually diagnosed by excluding other TMAs and DIC [51]. After the initiation of plasma exchange, if the baseline ADAMTS13 level is revealed to be more than 10% and STEC is negative, then the treatment should be switched to eculizumab as early as possible [53]. However, since the risk of *Neisseria meningitides* infection increases with eculizumab treatment, patients should be vaccinated or given a prophylactic antimicrobial agent appropriately. In the future, a definitive diagnosis is expected to be made based on the results of next-generation sequencing of the coding regions of complements [54].

#### Secondary thrombotic microangiopathy

- Pregnancy-related TMA

Thrombocytopenia develops in 5% to 10% of women during pregnancy or the postpartum period [55]. In most of the cases, it is an incidental change; however, it can also provide a clue to a coexisting systemic or gestational disorder. Acute fatty liver of pregnancy (AFLP) and preeclampsia/eclampsia/HELLP syndrome (hemolysis, elevated liver enzymes, low platelets) are representative of pregnancy-related TMA. DIC is another pregnancy-related coagulopathy, but the pathophysiologies of these two conditions differ. DIC is a complication of acute peripartum hemorrhage, placental abruption, retained stillbirth, and amniotic fluid embolism, and activated coagulation and subsequent consumptive coagulopathy are the basic mechanisms [56, 57]. HELLP syndrome is a severe complication of pre-eclampsia during pregnancy and occurs as a complication in 0.2% to 0.8% of pregnancies. Its pathogenesis is not fully understood, but it is thought to be associated with inadequate placentation secondary to a maternal immune response to invading trophoblasts [58]. HELLP is characterized by microvascular platelet thrombi, and the activation of the endothelium is thought to play a key role. Similar to TTP, the release of VWF multimers from activated endothelial cells and a reduction in ADAMTS13 activity result in increased amounts of the active form of VWF [59]. Thus, the clinical features of HELLP syndrome partially resemble those of TTP and HUS, and thrombocytopenia, MAHA, and hepatic damage are the major clinical symptoms. Thus, the typical clinical symptoms of HELLP syndrome begin with a pain in the right upper quadrant abdomen or epigastric pain, nausea, and vomiting. Since many patients with HELLP syndrome fit the diagnostic criteria for DIC, the differentiation of HELLP syndrome from DIC can be difficult [60]. Timely delivery is the standard management for HELLP syndrome, but if a patient shows sustained symptoms after delivery, the presence of other TMAs, such as aHUS, should be suspected.

AFLP is another rare but serious complication of pregnancy that often results in fulminant hepatic failure. Together with HELLP syndrome, liver diseases during pregnancy are accompanied by profound changes in the hemostatic system, including thrombocytopenia, decreased plasma levels of anticoagulants, and alterations in plasma levels of fibrinolysis. Decreased antithrombin levels have often been documented in this cohort and might be caused by a combination of liver failure and DIC [61]. The exact cause of AFLP is not yet elucidated, but the abnormal β-oxidation of fatty acids in fetal mitochondria caused by a genetic mutation in long-chain 3-hydroxyl coenzyme A dehydrogenase may contribute to the microvesicular fatty infiltration in the liver. The early termination of pregnancy is necessary, but the postpartum application of artificial liver support therapy has been recently proposed [62].

b. Collagen disease-associated TMA

The association of TMA with autoimmune diseases is a well-known fact, and systemic lupus erythematosus (SLE) has been described in up to 10% of TMA cases [63]. The pathogenesis of TMA in SLE is complicated, and complement over-activation via both classical and alternative pathways reportedly plays an important role in the pathogenesis of TMA in lupus nephritis [64]. Among SLE patients, kidney involvement is associated with a poor prognosis, and similar to other TMAs, plasma exchange, high-dose glucocorticoids, and intravenous immunoglobulin are the conventional treatments. Recently, the efficacy of rituximab has been reported [65]. A preliminary report regarding the use of eculizumab in SLE with lupus nephritis and the presence of TMA suggests its efficacy, but currently available evidence remains sparse [66].

c. Antiphospholipid syndrome/catastrophic antiphospholipid syndrome

Antiphospholipid syndrome (APS) is an acquired autoimmune thrombophilia defined by the development of (often multiple) venous and/or arterial thromboses and recurrent fetal losses in the presence of persistent antiphospholipid antibodies [67]. Among the antiphospholipid antibodies (lupus anticoagulant, anticardiolipin, and β2-glycoprotein I antibodies), the major player is considered to be β2-glycoprotein I at present. β2-glycoprotein I is a plasma protein that binds avidly to phospholipid surfaces [68]. Thrombocytopenia and a prolonged activated partial thromboplastin time (aPTT) can often be clues to APS. Recently, the usefulness of examining antibodies to prothrombin detected by directly coating prothrombin on irradiated ELISA plates was reported [69]. Antiplatelet and anticoagulant therapies are the treatments of choice for the prevention of thrombosis [70].

Catastrophic antiphospholipid syndrome (CAPS) is a rare variant that accounts for 1% of patients with APS but is highly fatal, with an estimated mortality rate of 50% [71]. CAPS is usually triggered by infection and trauma. Affected individuals are often children or young adults, and their clinical courses are characterized by the rapid onset of multifocal thrombosis associated with multi-organ failure upon presentation or developing rapidly over a course of days to weeks. Small vessel thrombosis, laboratory features of MAHA, and development of multisystem involvement within a very short period of time are the main characteristics of this syndrome. The mechanism of CAPS has not yet been clarified, but the involvement of an over-activated complement system is suspected. The treatment strategy is based on a combination of anticoagulation, glucocorticoids, and plasma exchange and/or intravenous immunoglobulin, so-called triple therapy. In refractory cases or in those with an initial life-threatening situation, immunosuppressive therapy using rituximab can be an effective option [72].

d. Transplant-associated TMA

Transplant-associated-TMA is a severe complication of hematopoietic cell transplantation, but it is also known to be associated with solid organ transplantation. The pathogenesis is poorly understood but may be related to endothelial injury induced by multifactorial causes including, graft versus host disease, infection, immune response, drug-induced effects, inflammatory cytokines release, and complement activation [73]. A high mortality rate has been documented for patients who are refractory to calcineurin inhibitor cessation. Recent evidence has linked transplant-associated TMA with aHUS, and eculizumab is expected to be effective for such conditions [74].

e. Malignancy-associated TMA

TMA can be a complication of certain malignancies [75]. TMA can also be induced by anticancer therapy [11]. Since the introduction of anti-vascular endothelial growth factor (VEGF) agents, the incidence of malignancy-associated TMA has increased dramatically to over 15% [76]. TMA is responsible for 15% of acute kidney failure cases in oncological settings, since the glomerular microvasculature is susceptible to injury from TMA. Anticancer-related TMA can be classified into two types: type I occurs secondary to chemotherapy (mitomycin C, gemcitabine) and is characterized by dose-dependent renal injury, while iatrogenic type II occurs secondary to anti-angiogenic agents and results in dose-independent renal involvement, with renal functional recovery typical after drug discontinuation [77, 78]. Recent research suggests that endothelial cell damage caused by the disrupted immunologic responses induced by immunosuppressive agents underlays. If the TMA has a refractory or relapsing clinical course and does not respond to plasmapheresis and steroids, immunosuppressive agents are the treatment of choice [77].

f. Drug-induced TMA

Other than anti-cancer agents, various drugs can cause TMA through dose-related toxic effects or immunologic reactions [79]. An immune mechanism of TMA was first described as a quinine-induced acute kidney injury induced by quinine-dependent antibodies causing endothelial damage and platelet activation [80]. Toxic mechanisms reported to be responsible for TMA include clopidogrel, gemcitabine, bevacizumab, and calcineurin inhibitors, such as cyclosporine and tacrolimus [81, 82]. Patients usually present with the sudden onset of anuric kidney injury, and symptoms of systemic illness often appear within hours after drug exposure. A thorough medical history can reveal prior exposure to a drug, and the identification of the causal drug and its discontinuation are essential for patient management. However, once drug-induced TMA occurs, supportive care is the only beneficial management.

### Heparin-induced thrombocytopenia

Heparin-induced thrombocytopenia (HIT) is a type of autoimmune disease caused by platelet-activating antibodies that recognize the multimolecular complexes of platelet factor 4 (PF4) bound to heparin. Since its core mechanism is autoantibody-induced platelet activation, patients often experience a high frequency of thrombotic events that can occur both in veins and arteries. Since heparins are commonly used in patients with sepsis for the prevention of deep vein thrombosis and catheter management [83], sepsis patients are at high-risk of developing HIT. The frequency of HIT is tenfold lower for low-molecular weight heparin (LMWH), compared with unfractionated heparin [84]. The clinical probability of HIT can be evaluated using clinical the clinical 4Ts scoring system (thrombocytopenia, timing of onset, thrombosis, and other causes of thrombocytopenia) [85]. A diagnosis of HIT can be supported by a positive PF4-dependent ELISA result plus a positive test for platelet-activating antibodies [86]. Most HIT episodes are triggered by proximate heparin exposure; however, the previous exposure cannot be identified in some cases [87]. Of note, HIT sometimes begins or worsens after the discontinuation of heparin, and diagnosis can be extremely difficult in such cases [88, 89]. In practice, HIT can be easily confused with sepsis-associated DIC, especially when it occurs in association with organ dysfunctions such as shock liver, adrenal hemorrhages, and skin/limb necrosis [90]. HIT typically persists for several weeks until the autoantibody diminishes. Anticoagulant therapy using argatroban, a direct thrombin inhibitor, is recommended. Activated partial thromboplastin time (APTT)-adjusted anticoagulant therapy ought to be effective, but patient management is not always easy [89].

### Immune thrombocytopenia purpura/Evans syndrome

Immune thrombocytopenic purpura (ITP) is an autoimmune disorder characterized by a low platelet count and mucocutaneous bleeding. It was previously called idiopathic thrombocytopenic purpura [91], but since thrombocytopenic purpura is mediated by autoantibodies, the “idiopathic” was amended to “immune” ITP can present as either a primary disorder (idiopathic) or a secondary disorder (induced by other conditions such as infection, lymphoproliferative disorders, and altered immune states). ITP can be induced by chronic viral or bacterial infection (*Helicobacter pylori*, HBV, and HIV), but infection is not necessarily present in many cases [92]. Its differentiation from sepsis-associated DIC is difficult if sepsis coexists. In some patients, a compensatory increase in platelet production is recognized, but impaired platelet production resulting from megakaryocytopoiesis inhibition is present in others. The decrease in platelets is caused by an increase in autoantibodies against self-antigens, particularly IgG antibodies against glycoprotein (GP) IIb/IIIa. Other responsible antibodies include those that react with GP Ib/IX, Ia/IIa, IV, and V, and the presence of antibodies against multiple antigens is typical [93]. However, since the detection of these antibodies is not common in clinical practice, the diagnosis of ITP continues to be based on exclusion. Antiplatelet antibodies mediate the accelerated clearance from the circulation mainly via the reticuloendothelial system [94]. Of note, since platelets are important for host defense, a low platelet count is a significant risk factor for infection in ITP patients [95].

Evans syndrome, a subtype of ITP, is characterized by the development of autoimmune hemolytic anemia (AIHA) and ITP. AIHA is commonly complicated by hemoglobinemia, acute kidney injury, and neurological disorder, making it difficult to differentiate from TMAs. Corticosteroids, intravenous immunoglobulin, and a splenectomy have been applied as initial treatments [96]; however, the emergence of effective drugs, such as rituximab and thrombopoietin-receptor agonists (eltrombopag and romiplostim), has significantly changed the management of ITP in the last decade [97, 98].

### Other circumferential diseases

#### Hemophagocytic syndrome

Hemophagocytic syndromes (HPSs), including hemophagocytic lymphohistiocytosis (HLH), are hyperinflammatory syndromes characterized by the excessive activation of macrophages, natural killer cells, and cytotoxic T cells. They can be genetic (primary) or acquired (secondary) and are potentially life-threatening. In the latter case, hyperinflammation is induced by large amounts of cytokines (tumor necrosis factor α, interferon-γ, interleukin-2, -6, etc.) released from activated macrophages and lymphocytes secondary to infection [99]. Secondary HPS occurs in the context of strong immunologic triggers such as Epstein Barr Virus infection, malignancy, autoimmune diseases, and drug hypersensitivity. Previously, the HLH-2004 criteria were most commonly used for diagnosis [100], and diagnoses were based on five criteria (fever, splenomegaly, bicytopenia, hypertriglyceridemia, and/or hypofibrinogenemia, and hemophagocytosis). More recently, three additional criteria have been introduced: low/absent natural killer cell-activity, hyperferritinemia, and high-soluble interleukin-2-receptor levels. The prompt treatment of the underlying causes is key to preventing irreversible tissue damage, but steroids and/or either etoposide-based or doxorubicin-based immunosuppressive therapy are the treatment choices for refractory cases [101].

#### Acute infectious purpura fulminans

Purpura fulminans, which is considered to be a thrombotic subtype of DIC, is a life-threatening condition characterized by sudden-onset progressive purpuric skin lesions and symmetrical acral necrosis [102]. Other than the typical skin and acral necrosis, purpura fulminans is characterized by fever, hemorrhage from multiple sites, and shock. It is classified into neonatal, idiopathic, and infection-induced (acute infectious purpura fulminans [AIPF]). AIPF is caused by various pathogens including *Neisseria meningitidis*, *Streptococcus pneumoniae*, *Haemophilus influenzae*, *Staphylococcus aureus*, and rickettsiae. The mechanism of purpura fulminans is poorly understood; however, a recent study revealed a severe deficiency in protein C caused by the loss of thrombomodulin on the endothelial surface during an early disease stage [103, 104]. Because of the strong association with DIC, laboratory tests show thrombocytopenia, a prolonged prothrombin time, an increased d-dimer level, and a decreasing fibrinogen level. Treatment for the underlying infection with broad-spectrum antibiotic agents is essential, and protein C supplementation is expected to be beneficial as an adjunctive therapy [14].

#### TAFRO syndrome

TAFRO is an acrostic for thrombocytopenia, anasarca (ascites, pleural effusion, etc.), fever, reticulin fibrosis (myelofibrosis), and organomegaly (and renal dysfunction). TAFRO syndrome is considered to be a subtype of Castleman’s disease, which is a lymphoproliferative disorder. An increased interleukin-6 level induced by human herpesvirus 8 infection has been identified as an important etiology of TAFRO syndrome [105]. As listed in its name, TAFRO is characterized by a constellation of symptoms and multiple lymphadenopathy of mild degree [106]. Three major criteria and at least one minor criterion and the exclusion of infectious, rheumatologic, and neoplastic diseases are required for the diagnosis of TAFRO [107]. Though autoantibodies are often detected, TAFRO should be discriminated from other autoimmune diseases such as SLE and rheumatoid arthritis. Patients are usually sensitive to steroids, and since a cytokine storm is deeply related to the pathogenesis of TAFRO, treatment with anti-interleukin-6 receptor antibody (tocilizumab) is expected to be useful [108].

#### Severe fever and thrombocytopenia syndrome

Severe fever and thrombocytopenia syndrome (SFTS) is a tick-borne infectious disease caused by the SFTS virus that has a wide spectrum of animal hosts, including livestock and wild animals. SFTS patients are specifically localized to East Asia, including China, Korea, and Japan [109]. The clinical features of SFTS include fever, thrombocytopenia, leukopenia, gastrointestinal symptoms, muscular symptoms, neurological abnormalities, and coagulopathy. SFTS is often accompanied by HPS, suggesting that dysregulated cytokine production and host immune responses play major roles in its pathogenesis. The histopathological findings are characterized by necrotizing lymphadenitis, with infiltration of the virus-infected cells to the local lymph nodes. The overall mortality rate has been reported to be 30% to 40% and is especially high in immunocompromised patients. Although there is no consensus regarding the ideal treatment for SFTS, several clinical trials have revealed the potential efficacy of plasma exchange with or without ribavirin, intravenous immunoglobulin, and corticosteroids [110].

#### Chronic liver disease

Thrombocytopenia is commonly seen in chronic liver disease such as liver cirrhosis. The mechanisms have been explained by hypersplenism, bone marrow suppression by hepatitis virus, and immunological removal of platelets. Recently, the decreased level of thrombopoietin has shed the new lights to the elucidation of a central mechanism. Thrombopoietin is produced mainly by the liver and the level is reduced along with the liver damage and leads to the thrombocytopenia. The treatment options include interventional partial splenic embolization and splenectomy. Thrombopoietin receptor agonists are an alternative choice for noninvasively treating thrombocytopenia [111].

Other than thrombocytopenia, hemostatic abnormalities, both favoring hemorrhage and favoring thrombosis, associated with chronic liver diseases. The mechanisms that cause hemorrhage include low platelet count, decreased levels of coagulation factors, vitamin K deficiency, and low levels of thrombin activatable fibrinolysis inhibitor. It had been thought that thrombotic events should be rarely happening but it has become evident that thrombotic complications can occur in cirrhotic patients. The mechanisms are explained by elevated levels of factor VIII and VWF, decreased levels of protein C, protein S, antithrombin, and decreased levels of plasminogen [112].

### Diagnostic algorithm

**Fig. 1:**
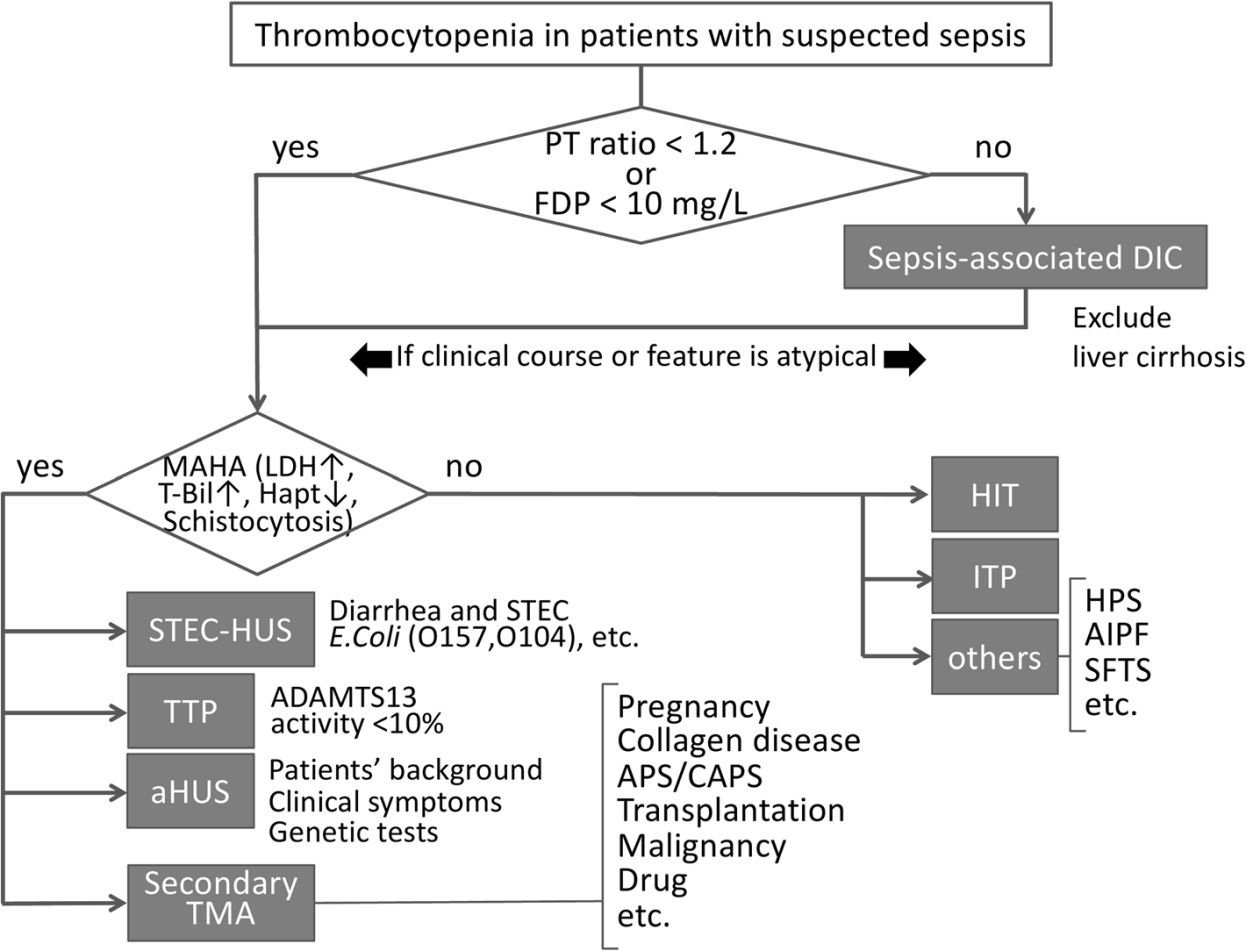
An algorithm to differentiate sepsis-associated DIC from other diseases with thrombocytopenia. Both the prothrombin time (PT) ratio and the level of fibrin/fibrinogen degradation products are elevated in sepsis-associated DIC (disseminated intravascular coagulation). If either marker is within the normal range, other diseases can be suspected. If microangiopathic hemolytic anemia (MAHA) is recognized, *Escherichia coli* (STEC)-hemolytic uremic syndrome (HUS) will be discriminated by performing a stool culture or polymerase chain reaction (PCR) assay first. If it is not, thrombotic thrombocytopenic purpura (TTP), atypical HUS (aHUS), or secondary thrombotic microangiopathy are suspicious, and the early initiation of plasma exchange is recommended unless a disintegrin and metalloproteinase with a thrombospondin type 1 motif, member 13 (ADAMTS13) level is confirmed. TTP is diagnosed by the identification of a low ADAMTS13 activity (< 10%). If plasma exchange is ineffective, refractory TTP, aHUS, or other disease is suspicious and the use of either rituximab or eculizumab will be considered. In those cases, the laboratory findings and clinical symptoms such as acute kidney injury and gastrointestinal or neurological damage will be carefully examined; if these findings suggest aHUS, the patient’s age and medical and family histories can be helpful for a diagnosis. Similarly, the possibility of secondary TMAs can be considered. If the presence of MAHA is not recognized, the possibility of other diseases such as heparin-induced thrombocytopenia (HIT), immune thrombocytopenia purpura (ITP), hemophagocytic syndrome (HPS), acute infectious purpura fulminans (AIPF), severe fever and thrombocytopenia syndrome (SFTS), and thrombocytopenia, anasarca, fever, reticulin fibrosis, and organomegaly (TAFRO) syndrome would be considered

**Fig. 2:**
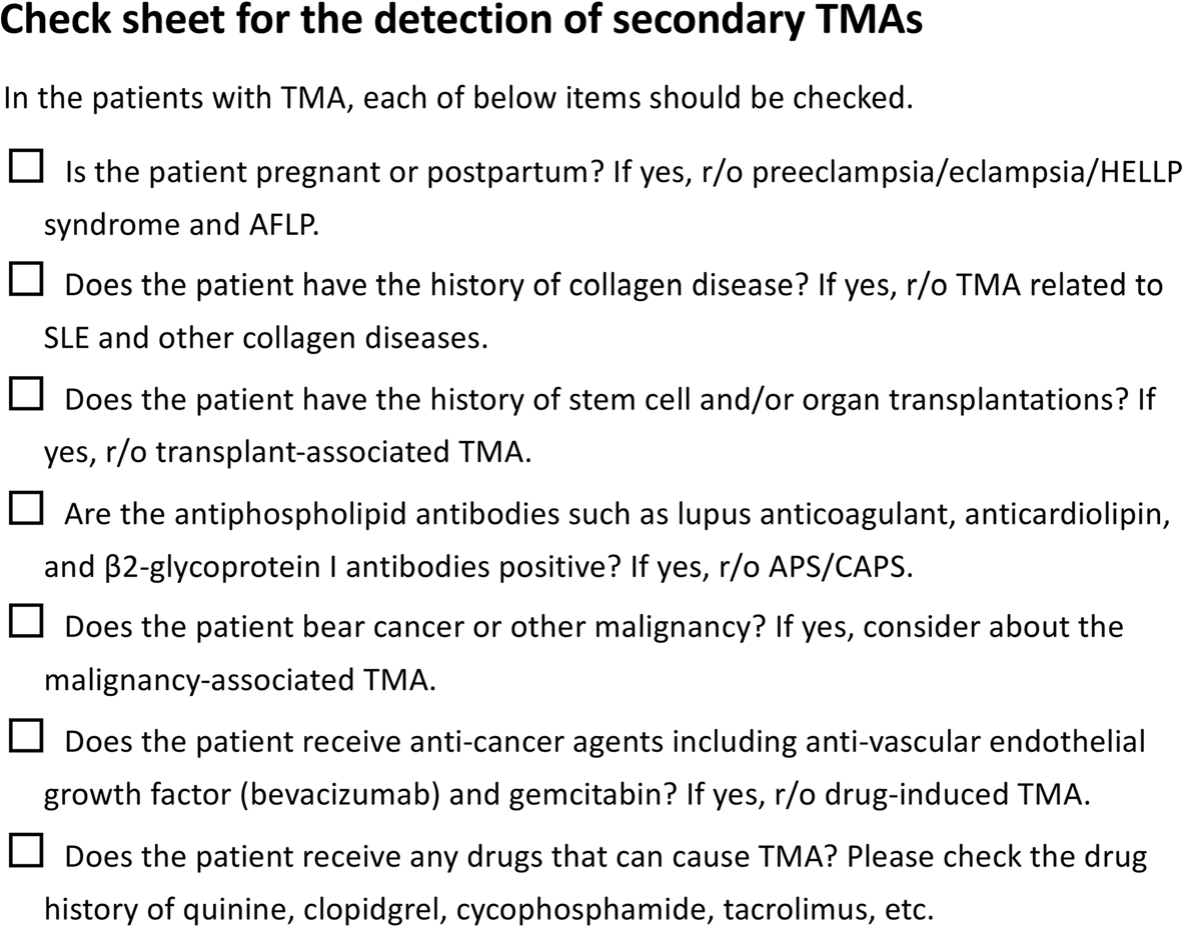
Check sheet for the secondary thrombotic microangiopathic diseases. For the discrimination in secondary TMA, each item in the check sheet will be confirmed

A provisional diagnostic algorithm was constructed based on the above review (Fig. 1). First, when patients exhibit thrombocytopenia (platelet count < 150, × 10^9^ L^−1^, both the PT time and the level of fibrin/fibrinogen degradation products are measured. If either biomarker has a normal value (PT ratio < 1.2 and/or fibrinogen/fibrin degradation products < 10 mg/L), the possibility of diseases other than sepsis-associated DIC should be considered. According the previous survey, the prevalence of thrombocytopenia with insignificant FDP elevation or insignificant PT prolongation were as follow: 68 cases out of 500 patients who were diagnosed as having DIC (Japanese Association for Acute Medicine [JAAM] criteria) (13.6%) showed FDP level of less than 10 mg/mL, and 91 cases out of 500 JAAM-DIC patients (18.2%) showed PT ratio of less than 1.2. Of note, even if the PT ratio and the level of fibrin/fibrinogen degradation products are elevated, the possible coexistence of other diseases should be considered when the changes in patients’ coagulation status do not correlate with their clinical courses of infection. If patients have a normal PT ratio or level of fibrin/fibrinogen degradation products but show the findings of MAHA, i.e., elevated levels of LDH, total bilirubin, decreased haptoglobin, and schistocytosis, the presence of TMA is suspected. The patient should be evaluated for presence of STEC-HUS by assessing symptoms of enterocolitis and by examining for presence of STEC using a culture-based assay, serological assessment, or polymerase chain reaction. While, if the patients do not show abdominal symptoms and fever, aHUS, TTP, and secondary TMAs are differentiated. With respect to the usefulness of this algorithm, since this diagnostic approach has just been proposed, the validation should be performed in the future study. When the presence of MAHA is ruled out, diseases other than TMA, such as HIT, ITP, and others, are the candidates for the consideration; if the presence of MAHA is confirmed, the likelihood of TMAs is high and the initiation of plasma exchange will be considered. In severe cases, plasma exchange is recommended to be initiated within 4–8 h; however, if the response is not sufficient, diseases other than TTP, such as aHUS or secondary TMA, will be suspected again. For the discrimination in secondary TMA, the use of check sheet is helpful (Fig. 2). The clinical features and initial therapies for the various thrombocytopenic diseases are summarized in the Table 1.

**Table 1:** Comparison of sepsis-associated DIC and circumferential diseases

Category	Disease	Cause	Clinical features	Treatment
DIC		Infection-induced expression of tissue factor and phosphatidylserine of the cellular membrane	Thrombotic phenotype of coagulation disorder with fibrinolysis suppression	Management of infectious focus, potentially anticoagulant therapy
TMA	TTP (acquired)	Autoantibody inhibition of ADAMTS13 activity	TTP pentad (thrombocytopenia, MAHA, fluctuating neurological signs, renal impairment and fever)	Plasma exchange, immunosuppressive therapy, recombinant ADAMTS13 if possible
	STEC-HUS	Shiga toxin-producing *Escherichia coli*	Hemorrhagic enterocolitis, fever, thrombocytopenia, MAHA, acute kidney injury	Avoiding antibiotic therapy and supportive care
	aHUS	Uncontrolled activity of alternative complement pathway.	Thrombocytopenia, MAHA, acute kidney injury	Plasma exchange, and anti-C5 monoclonal antibody (eculizumab)
Secondary TMA	HELLP syndrome	Inadequate placentation secondary to maternal immune response to invading trophoblast.	Hemolysis, elevated liver enzymes, and low platelets	Timely delivery
	APS/CAPS (primary)	Antiphospholipid antibodies (β2-glycoprotein I)	Multiple venous and arterial thrombosis, repeated miscarriage, multi-organ failure (CAPS)	Anticoagulation, glucocorticoids, plasma exchange, IVIg
HIT		Platelet-activating antibodies to platelet factor 4 bound to heparin	4Ts scoring system (thrombocytopenia, the timing of onset, thrombosis, and other causes of thrombocytopenia)	Discontinuation of heparin and anticoagulant therapy by argatroban
ITP		IgG antibodies against GP IIb/IIIa, Ia/IIa, IV, and V	Thrombocytopenia (with AIHA, kidney injury, and neurological disorder [Evans syndrome])	Thrombopoietin-receptor agonists (eltrombopag and romiplostim), steroid, IVIg, splenectomy
Others	HPS (acquired)	Over-produced cytokines triggered commonly by infection	Fever, splenomegaly, bicytopenia, hypertriglyceridemia and/or hypofibrinogenemia, and hemophagocytosis	Treatment for the underlying cause, steroid, immunosuppressive therapy
	AIPF	Bacteria or rickettsiae infection-induced protein C deficiency	Purpura, symmetrical acral necrosis, fever, hemorrhage, and shock	Treatment for the underlying infection, protein C supplementation
	TAFRO	Human herpesvirus 8 infection-induced interleukin-6 elevation	Thrombocytopenia, anasarca, fever, reticulin fibrosis, organomegaly	Steroid, anti-interleukin-6 receptor antibody (tocilizumab)
	SFTS	Tick-borne SFTS virus infection	Fever, thrombocytopenia, leukopenia, gastrointestinal symptoms, muscular symptoms, neurological abnormalities, coagulopathy	Plasma exchange, ribavirin, IVIg, steroid

*DIC* disseminated intravascular coagulation, *TMA* thrombotic microangiopathy, *TTP* thrombotic thrombocytopenic purpura, *ADAMTS13* a disintegrin and metalloproteinase with a thrombospondin type 1 motif, member 13, *MAHA* microangiopathic hemolytic anemia, *STEC* Shiga toxin-producing *Escherichia coli*, *HUS* hemolytic uremic syndrome, *aHUS* atypical HUS, *HELLP* hemolysis, elevated liver enzymes low platelets, *APS* antiphospholipid syndrome, *CAPS* catastrophic antiphospholipid syndrome, *IVIg* intravenous immunoglobulin, *HIT* heparin-induced thrombocytopenia, *ITP* immune thrombocytopenia purpura, *HPS* hemophagocytic syndromes, *AIPF* acute infectious purpura fulminans, *SFTS* fever and thrombocytopenia syndrome

## Conclusions

Besides DIC, thrombocytopenia can occur in response to various backgrounds in patients with sepsis. For the appropriate management of thrombocytopenia, early diagnosis and the urgent initiation of proper treatments are crucial. Therefore, we have proposed a new diagnostic algorithm that enables a rapid diagnosis. Since recent advances in diagnostic tests and therapeutics have been remarkable, it is now mandatory to recognize the pathophysiology of individual diseases correctly. We also emphasize that we have to keep in mind that since most of the differential diseases are quite rare, the high-quality evidence that supports the therapeutics is still lacking. Future study is warranted in this area. This review summarized the pathogenic and clinical features of the major thrombocytopenic conditions.

## Abbreviations

ADAMTS13: A disintegrin and metalloproteinase with a thrombospondin type 1 motif, member 13
AFLP: Acute fatty liver of pregnancy
AIHA: Autoimmune hemolytic anemia
AIPF: Acute infectious purpura fulminans
APS: Antiphospholipid syndrome
APTT: Activated partial thromboplastin time
CAPS: Catastrophic antiphospholipid syndrome
DAMPs: Damage-associated molecular patterns
DIC: Disseminated intravascular coagulation
GP: Glycoprotein
HELLP: Hemolysis, elevated liver enzymes low platelets
HIT: Heparin-induced thrombocytopenia
HLH: Hemophagocytic lymphohistiocytosis
HMGB1: High-mobility group box 1
HPSs: Hemophagocytic syndromes
HUS: Hemolytic uremic syndrome
ISTH: International Society for Thrombosis and Hemostasis
ITP: Immune thrombocytopenic purpura
LMWH: Low molecular weight heparin
MAHA: Microangiopathic hemolytic anemia
NETs: Neutrophil extracellular traps
PAI-1: Plasminogen activator inhibitor-1
PAMPs: Pathogen-associated molecular patterns
PARs: Protease-activated receptors
PT: Prothrombin time
SFTS: Severe fever and thrombocytopenia syndrome
SLE: Systemic lupus erythematosus
SOFA: Sequential Organ Failure Assessment
STEC: Shiga toxin-producing *Escherichia coli*
TAFRO: Thrombocytopenia, anasarca, fever, reticulin fibrosis and organomegaly
TMA: Thrombotic microangiopathy
TTP: Thrombotic thrombocytopenic purpura
VEGF: Vascular endothelial growth factor
VWF: Von Willebrand factor

## References

[1] Singer M, Deutschman CS, Seymour CW, Shankar-Hari M, Annane D, Bauer M, Bellomo R, Bernard GR, Chiche JD, Coopersmith CM, Hotchkiss RS, Levy MM, Marshall JC, Martin GS, Opal SM, Rubenfeld GD, van der Poll T, Vincent JL, Angus DC (2016). The Third International Consensus Definitions for Sepsis and Septic Shock (Sepsis-3). JAMA..

[2] Howell MD, Davis AM (2017). Management of sepsis and septic shock. JAMA..

[3] Thiery-Antier N, Binquet C, Vinault S, Meziani F, Boisramé-Helms J, Quenot JP (2016). Is thrombocytopenia an early prognostic marker in septic shock?. Crit Care Med.

[4] Claushuis TA, van Vught LA, Scicluna BP, Wiewel MA, Klein Klouwenberg PM, Hoogendijk AJ, Ong DS, Cremer OL, Horn J, Franitza M, Toliat MR, Nürnberg P, Zwinderman AH, Bonten MJ, Schultz MJ, van der Poll T (2016). Thrombocytopenia is associated with a dysregulated host response in critically ill sepsispatients. Blood..

[5] de Stoppelaar SF, van’t Veer C, van der Poll T (2014). The role of platelets in sepsis. Thromb Haemost.

[6] Iba T, Levy JH (2018). Inflammation and thrombosis: roles of neutrophils, platelets and endothelial cells and their interactions in thrombus formation during sepsis. J Thromb Haemost.

[7] Bermejo-Martin JF, Martín-Fernandez M, López-Mestanza C, Duque P, Almansa R (2018). Shared features of endothelial dysfunction between sepsis and its preceding risk factors. J Clin Med.

[8] Nishida O, Ogura H, Egi M, Fujishima S, Hayashi Y, Iba T, Imaizumi H, Inoue S, Kakihana Y, Kotani J, Kushimoto S, Masuda Y, Matsuda N, Matsushima A, Nakada TA, Nakagawa S, Nunomiya S, Sadahiro T, Shime N, Yatabe T, Hara Y, Hayashida K, Kondo Y, Sumi Y, Yasuda H, Aoyama K, Azuhata T, Doi K, Doi M, Fujimura N, Fuke R, Fukuda T, Goto K, Hasegawa R, Hashimoto S, Hatakeyama J, Hayakawa M, Hifumi T, Higashibeppu N, Hirai K, Hirose T, Ide K, Kaizuka Y, Kan'o T, Kawasaki T, Kuroda H, Matsuda A, Matsumoto S, Nagae M, Onodera M, Ohnuma T, Oshima K, Saito N, Sakamoto S, Sakuraya M, Sasano M, Sato N, Sawamura A, Shimizu K, Shirai K, Takei T, Takeuchi M, Takimoto K, Taniguchi T, Tatsumi H, Tsuruta R, Yama N, Yamakawa K, Yamashita C, Yamashita K, Yoshida T, Tanaka H, Oda S (2018). The Japanese Clinical Practice Guidelines for Management of Sepsis and Septic Shock 2016 (J-SSCG 2016). Acute Med Surg.

[9] Iba T, Levy JH, Wada H, Thachil J, Warkentin TE, Levi M. Differential diagnoses for sepsis-induced disseminated intravascular coagulation. J Thromb Haemost. 2018. 10.1111/jth.14354. 10.1111/jth.14354 30618150

[10] Nguyen TC, Cruz MA, Carcillo JA (2015). Thrombocytopenia-associated multiple organ failure and acute kidney injury. Crit Care Clin.

[11] George JN, Nester CM (2014). Syndromes of thrombotic microangiopathy. N Engl J Med.

[12] Ruggenenti P, Noris M, Remuzzi G (2001). Thrombotic microangiopathy, hemolytic uremic syndrome, and thrombotic thrombocytopenic purpura. Kidney Int.

[13] Taylor FB, Toh CH, Hoots WK, Wada H, Levi M (2001). Towards definition, clinical and laboratory criteria, and a scoring system for disseminated intravascular coagulation. Thromb Haemost.

[14] Gando S, Iba T, Eguchi Y, Ohtomo Y, Okamoto K, Koseki K, Mayumi T, Murata A, Ikeda T, Ishikura H, Ueyama M, Ogura H, Kushimoto S, Saitoh D, Endo S, Shimazaki S (2006). A multicenter, prospective validation of disseminated intravascular coagulation diagnostic criteria for critically ill patients: comparing current criteria. Crit Care Med.

[15] Vincent JL, Castro P, Hunt BJ, Jörres A, Praga M, Rojas-Suarez J, Watanabe E (2018). Thrombocytopenia in the ICU: disseminated intravascular coagulation and thrombotic microangiopathies-what intensivists need to know. Crit Care.

[16] Semeraro N, Ammollo CT, Semeraro F, Colucci M (2012). Sepsis, thrombosis and organ dysfunction. Thromb Res.

[17] Liaw PC, Ito T, Iba T, Thachil J, Zeerleder S (2016). DAMP and DIC: The role of extracellular DNA and DNA-binding proteins in the pathogenesis of DIC. Blood Rev.

[18] Gando S, Saitoh D, Ogura H, Mayumi T, Koseki K, Ikeda T, Ishikura H, Iba T, Ueyama M, Eguchi Y, Ohtomo Y, Okamoto K, Kushimoto S, Endo S, Shimazaki S (2008). Natural history of disseminated intravascular coagulation diagnosed based on the newly established diagnostic criteria for critically ill patients: results of a multicenter, prospective survey. Crit Care Med.

[19] Semeraro N, Ammollo CT, Semeraro F, Colucci M (2015). Coagulopathy of acute Sepsis. Semin Thromb Hemost.

[20] Engelmann B, Massberg S (2013). Thrombosis as an intravascular effector of innate immunity. Nat Rev Immunol.

[21] Corrigan JJ, Ray WL, May N (1968). Changes in the blood coagulation system associated with septicemia. N Engl J Med.

[22] Østerud B, Bjørklid E (2001). The tissue factor pathway in disseminated intravascular coagulation. Semin Thromb Hemost.

[23] Nieman MT (2016). Protease-activated receptors in hemostasis. Blood..

[24] Ma R, Xie R, Yu C, Si Y, Wu X, Zhao L, Yao Z, Fang S, Chen H, Novakovic V, Gao C, Kou J, Bi Y, Thatte HS, Yu B, Yang S, Zhou J, Shi J (2017). Phosphatidylserine-mediated platelet clearance by endothelium decreases platelet aggregates and procoagulant activity in sepsis. Sci Rep.

[25] Levi M, van der Poll T (2017). Coagulation and sepsis. Thromb Res.

[26] Moake JL (2002). Thrombotic microangiopathies. N Engl J Med.

[27] Scully M, Hunt BJ, Benjamin S, Liesner R, Rose P, Peyvandi F, Cheung B, Machin SJ (2012). Guidelines on the diagnosis and management of thrombotic thrombocytopenic purpura and other thrombotic microangiopathies. Br J Haematol.

[28] Wada H, Matsumoto T, Suzuki K, Imai H, Katayama N, Iba T, Matsumoto M (2018). Differences and similarities between disseminated intravascular coagulation and thrombotic microangiopathy. Thromb J.

[29] Thomas W, Cutler JA, Moore GW, McDonald V, Hunt BJ. The utility of a fast turnaround ADAMTS13 activity in the diagnosis and exclusion of thrombotic thrombocytopenic purpura. Br J Haematol. 2018. 10.1111/bjh.15219. 10.1111/bjh.15219 29687882

[30] Levi M, Scully M, Singer M (2018). The role of ADAMTS-13 in the coagulopathy of sepsis. J Thromb Haemost.

[31] Groot E, Fijnheer R, Sebastian SA, de Groot PG, Lenting PJ (2009). The active conformation of von Willebrand factor in patients with thrombotic thrombocytopenic purpura in remission. J Thromb Haemost.

[32] Kremer Hovinga JA, Coppo P, Lämmle B, Moake JL, Miyata T, Vanhoorelbeke K (2017). Thrombotic thrombocytopenic purpura. Nat Rev Dis Primers.

[33] Wada H, Matsumoto T, Hatada T (2012). Diagnostic criteria and laboratory tests for disseminated intravascular coagulation. Expert Rev Hematol.

[34] Wada H, Matsumoto T, Yamashita Y (2014). Natural history of thrombotic thrombocytopenic purpura and hemolytic uremic syndrome. Semin Thromb Hemost.

[35] Iba T, Gando S, Thachil J (2014). Anticoagulant therapy for sepsis-associated disseminated intravascular coagulation: the view from Japan. J Thromb Haemost.

[36] Peyvandi F, Scully M, Kremer Hovinga JA, Cataland S, Knöbl P, Wu H, Artoni A, Westwood JP, Mansouri Taleghani M, Jilma B, Callewaert F, Ulrichts H, Duby C, Tersago D (2016). Caplacizumab for acquired thrombotic thrombocytopenic purpura. N Engl J Med.

[37] Scully M, Cataland SR, Peyvandi F, Coppo P, Knöbl P, Kremer Hovinga JA, Metjian A, de la Rubia J, Pavenski K, Callewaert F, Biswas D, De Winter H, Zeldin RK (2019). Caplacizumab treatment for acquired thrombotic thrombocytopenic purpura. N Engl J Med.

[38] Tersteeg C, Schiviz A, De Meyer SF, Plaimauer B, Scheiflinger F, Rottensteiner H, Vanhoorelbeke K (2015). Potential for recombinant ADAMTS13 as an effective therapy for acquired thrombotic thrombocytopenic purpura. Arterioscler Thromb Vasc Biol.

[39] Talarico V, Aloe M, Monzani A, Miniero R, Bona G (2016). Hemolytic uremic syndrome in children. Minerva Pediatr.

[40] Karmali MA (2018). Factors in the emergence of serious human infections associated with highly pathogenic strains of shiga toxin-producing Escherichia coli. Int J Med Microbiol.

[41] Ingelbeen B, Bruyand M, Mariani-Kurkjian P, Le Hello S, Danis K, Sommen C, Bonacorsi S, de Valk H (2018). Emerging Shiga-toxin-producing Escherichia coli serogroup O80 associated hemolytic and uremic syndrome in France, 2013-2016: differences with other serogroups. PLoS One.

[42] Liu F, Huang J, Sadler JE (2011). Shiga toxin (Stx)1B and Stx2B induce von Willebrand factor secretion from human umbilical vein endothelial cells through different signaling pathways. Blood..

[43] Noris M, Mescia F, Remuzzi G (2012). STEC-HUS, atypical HUS and TTP are all diseases of complement activation. Nat Rev Nephrol.

[44] Kielstein JT, Beutel G, Fleig S, Steinhoff J, Meyer TN, Hafer C, Kuhlmann U, Bramstedt J, Panzer U, Vischedyk M, Busch V, Ries W, Mitzner S, Mees S, Stracke S, Nürnberger J, Gerke P, Wiesner M, Sucke B, Abu-Tair M, Kribben A, Klause N, Schindler R, Merkel F, Schnatter S, Dorresteijn EM, Samuelsson O, Brunkhorst R (2012). Best supportive care and therapeutic plasma exchange with or without eculizumab in Shiga-toxin-producing E. coli O104:H4 induced haemolytic-uraemic syndrome: an analysis of the German STEC-HUS registry. Nephrol Dial Transplant.

[45] Jokiranta TS (2017). HUS and atypical HUS. Blood..

[46] Spinale JM, Ruebner RL, Kaplan BS, Copelovitch L (2013). Update on Streptococcus pneumoniae associated hemolytic uremic syndrome. Curr Opin Pediatr.

[47] Meinel C, Spartà G, Dahse HM, Hörhold F, König R, Westermann M, Coldewey SM, Cseresnyés Z, Figge MT, Hammerschmidt S, Skerka C, Zipfel PF (2018). Streptococcus pneumoniae from patients with hemolytic uremic syndrome binds human plasminogen via the surface protein PspC and uses plasmin to damage human endothelial cells. J Infect Dis.

[48] Azoulay E, Knoebl P, Garnacho-Montero J, Rusinova K, Galstian G, Eggimann P, Abroug F, Benoit D, von Bergwelt-Baildon M, Wendon J, Scully M (2017). Expert statements on the standard of care in critically ill adult patients with atypical hemolytic uremic syndrome. Chest..

[49] Nester CM, Thomas CP (2012). Atypical hemolytic uremic syndrome: what is it, how is it diagnosed, and how is it treated?. Hematology Am Soc Hematol Educ Program..

[50] Noris M, Caprioli J, Bresin E (2010). Relative role of genetic complement abnormalities in sporadic and familial aHUS and their impact on clinical phenotype. Clin J Am Soc Nephrol.

[51] Scully M, Goodship T (2014). How I treat thrombotic thrombocytopenic purpura and atypical haemolytic uraemic syndrome. Br J Haematol.

[52] Fujisawa M, Kato H, Yoshida Y, Usui T, Takata M, Fujimoto M, Wada H, Uchida Y, Kokame K, Matsumoto M, Fujimura Y, Miyata T, Nangaku M (2018). Clinical characteristics and genetic backgrounds of Japanese patients with atypical hemolyticuremic syndrome. Clin Exp Nephrol.

[53] Legendre CM, Licht C, Muus P, Greenbaum LA, Babu S, Bedrosian C, Bingham C, Cohen DJ, Delmas Y, Douglas K, Eitner F, Feldkamp T, Fouque D, Furman RR, Gaber O, Herthelius M, Hourmant M, Karpman D, Lebranchu Y, Mariat C (2013). Terminal complement inhibitor eculizumab in atypical hemolytic- uremic syndrome. N Engl J Med.

[54] Larsen CP, Wilson JD, Best-Rocha A, Beggs ML, Hennigar RA (2018). Genetic testing of complement and coagulation pathways in patients with severe hypertension and renal microangiopathy. Mod Pathol.

[55] Cines DB, Levine LD (2017). Thrombocytopenia in pregnancy. Hematology Am Soc Hematol Educ Program..

[56] Thomas MR, Robinson S, Scully MA (2016). How we manage thrombotic microangiopathies in pregnancy. Br J Haematol.

[57] Erez O (2017). Disseminated intravascular coagulation in pregnancy-clinical phenotypes and diagnostic scores. Thromb Res.

[58] Abildgaard U, Heimdal K (2013). Pathogenesis of the syndrome of hemolysis, elevated liver enzymes, and low platelet count (HELLP): a review. Eur J Obstet Gynecol Reprod Biol.

[59] Hulstein JJ, van Runnard Heimel PJ, Franx A, Lenting PJ, Bruinse HW, Silence K, de Groot PG, Fijnheer R (2006). Acute activation of the endothelium results in increased levels of active von Willebrand factor in hemolysis, elevated liver enzymes and low platelets (HELLP) syndrome. J Thromb Haemost.

[60] Haram K, Mortensen JH, Mastrolia SA, Erez O (2017). Disseminated intravascular coagulation in the HELLP syndrome: how much do we really know?. J Matern Fetal Neonatal Med.

[61] Lamprecht A, Morton A, Laurie J, Lee W (2018). Acute fatty liver of pregnancy and concomitant medical conditions: a review of cases at a quaternary obstetric hospital. Obstet Med.

[62] Wu Z, Huang P, Gong Y, Wan J, Zou W (2018). Treating acute fatty liver of pregnancy with artificial liver support therapy: Systematic review. Medicine..

[63] de Holanda MI, Pôrto LC, Wagner T, Christiani LF, Palma LMP (2017). Use of eculizumab in a systemic lupus erythemathosus patient presenting thrombotic microangiopathy and heterozygous deletion in CFHR1-CFHR3. A case report and systematic review. Clin Rheumatol.

[64] Song D, Wu LH, Wang FM, Yang XW, Zhu D, Chen M, Yu F, Liu G, Zhao MH (2013). The spectrum of renal thrombotic microangiopathy in lupus nephritis. Arthritis Res Ther.

[65] Sun F, Wang X, Wu W, Wang K, Chen Z, Li T, Ye S (2018). TMA secondary to SLE: rituximab improves overall but not renal survival. Clin Rheumatol.

[66] Sciascia S, Radin M, Yazdany J, Tektonidou M, Cecchi I, Roccatello D, Dall'Era M (2017). Expanding the therapeutic options for renal involvement in lupus: eculizumab, available evidence. Rheumatol Int.

[67] Groot N, de Graeff N, Avcin T, Bader-Meunier B, Dolezalova P, Feldman B, Kenet G, Koné-Paut I, Lahdenne P, Marks SD, McCann L, Pilkington CA, Ravelli A, van Royen-Kerkhof A, Uziel Y, Vastert SJ, Wulffraat NM, Ozen S, Brogan P, Kamphuis S, Beresford MW (2017). European evidence-based recommendations for diagnosis and treatment of paediatric antiphospholipid syndrome: the SHARE initiative. Ann Rheum Dis.

[68] Garcia D, Erkan D (2018). Diagnosis and management of the antiphospholipid syndrome. N Engl J Med.

[69] Hoxha A, Mattia E, Tonello M, Grava C, Pengo V, Ruffatti A (2017). Antiphosphatidylserine/prothrombin antibodies as biomarkers to identify severe primary antiphospholipid syndrome. Clin Chem Lab Med.

[70] Sciascia S, Sanna G, Murru V, Roccatello D, Khamashta MA, Bertolaccini ML (2014). Anti-prothrombin (aPT) and anti-phosphatidylserine/prothrombin (aPS/PT) antibodies and the risk of thrombosis in the antiphospholipid syndrome. A systematic review. Thromb Haemost.

[71] Espinosa G, Rodríguez-Pintó I, Cervera R (2017). Catastrophic antiphospholipid syndrome: an update. Panminerva Med.

[72] Legault K, Schunemann H, Hillis C, Yeung C, Akl EA, Carrier M, Cervera R, Crowther M, Dentali F, Erkan D, Espinosa G, Khamashta M, Meerpohl JJ, Moffat K, O'Brien S, Pengo V, Rand JH, Rodriguez Pinto I, Thom L, Iorio A. McMaster RARE-Bestpractices clinical practice guideline on diagnosis and management of the catastrophic antiphospholipid syndrome. J Thromb Haemost. 2018. 10.1111/jth.14192. 10.1111/jth.14192 29978552

[73] Zeisbrich M, Becker N, Benner A, Radujkovic A, Schmitt K, Beimler J, Ho AD, Zeier M, Dreger P, Luft T (2017). Transplant-associated thrombotic microangiopathy is an endothelial complication associated with refractoriness of acute GvHD. Bone Marrow Transplant.

[74] Gavriilaki E, Sakellari I, Anagnostopoulos A, Brodsky RA (2017). Transplant-associated thrombotic microangiopathy: opening Pandora's box. Bone Marrow Transplant.

[75] Morton JM, George JN (2016). Microangiopathic hemolytic anemia and thrombocytopenia in patients with cancer. J Oncol Pract.

[76] Izzedine H, Perazella MA (2015). Thrombotic microangiopathy, cancer, and cancer drugs. Am J Kidney Dis.

[77] Kheder El-Fekih R, Deltombe C, Izzedine H (2017). Thrombotic microangiopathy and cancer. Nephrol Ther.

[78] Eremina V, Jefferson JA, Kowalewska J, Hochster H, Haas M, Weisstuch J, Richardson C, Kopp JB, Kabir MG, Backx PH, Gerber HP, Ferrara N, Barisoni L, Alpers CE, Quaggin SE (2008). VEGF inhibition and renal thrombotic microangiopathy. N Engl J Med.

[79] Al-Nouri ZL, Reese JA, Terrell DR, Vesely SK, George JN (2015). Drug-induced thrombotic microangiopathy: a systematic review of published reports. Blood..

[80] Gottschall JL, Neahring B, McFarland JG, Wu GG, Weitekamp LA, Aster RH (1994). Quinine-induced immune thrombocytopenia with hemolytic uremic syndrome: clinical and serological findings in nine patients and review of literature. Am J Hematol.

[81] Medina PJ, Sipols JM, George JN (2001). Drug-associated thrombotic thrombocytopenic purpura-hemolytic uremic syndrome. Curr Opin Hematol.

[82] Dlott JS, Danielson CF, Blue-Hnidy DE, McCarthy LJ (2004). Drug-induced thrombotic thrombocytopenic purpura/hemolytic uremic syndrome: a concise review. Ther Apher Dial.

[83] Kleinpell R, Aitken L, Schorr CA (2013). Implications of the new international sepsis guidelines or nursing care. Am J Crit Care.

[84] Martel N, Lee J, Wells PS (2005). Risk for heparin-induced thrombocytopenia with unfractionated and low-molecular-weight heparin thromboprophylaxis: a meta-analysis. Blood..

[85] Warkentin TE (2016). Clinical picture of heparin-induced thrombocytopenia (HIT) and its differentiation from non-HIT thrombocytopenia. Thromb Haemost.

[86] Warkentin TE, Greinacher A, Gruel Y, Aster RH, Chong BH (2011). Scientific and Standardization Committee of the International Society on Thrombosis and Haemostasis. Laboratory testing for heparin-induced thrombocytopenia: a conceptual framework and implications for diagnosis. J Thromb Haemost.

[87] Poudel DR, Ghimire S, Dhital R, Forman D, Warkentin TE (2017). Spontaneous HIT syndrome post-knee replacement surgery with delayed recovery of thrombocytopenia: a case report and literature review. Platelets..

[88] Warkentin TE, Greinacher A (2016). Management of heparin-induced thrombocytopenia. Curr Opin Hematol.

[89] Greinacher A, Selleng K, Warkentin TE (2017). Autoimmune heparin-induced thrombocytopenia. J Thromb Haemost.

[90] Warkentin TE (2015). Ischemic limb gangrene with pulses. N Engl J Med.

[91] Rodeghiero F, Stasi R, Gernsheimer T, Michel M, Provan D, Arnold DM, Bussel JB, Cines DB, Chong BH, Cooper N, Godeau B, Lechner K, Mazzucconi MG, McMillan R, Sanz MA, Imbach P, Blanchette V, Kühne T, Ruggeri M, George JN (2009). Standardization of terminology, definitions and outcome criteria in immune thrombocytopenic purpura of adults and children: report from an international working group. Blood..

[92] Liebman HA (2009). Recognizing and treating secondary immune thrombocytopenic purpura associated with lymphoproliferative disorders. Semin Hematol.

[93] Cines DB, Blanchette VS (2002). Immune thrombocytopenic purpura. N Engl J Med.

[94] Johnsen J (2012). Pathogenesis in immune thrombocytopenia: new insights. Hematology Am Soc Hematol Educ Program..

[95] Qu M, Liu Q, Zhao HG, Peng J, Ni H, Hou M, Jansen AJG (2018). Low platelet count as risk factor for infections in patients with primary immune thrombocytopenia: a retrospective evaluation. Ann Hematol.

[96] Neunert CE, Cooper N (2018). Evidence-based management of immune thrombocytopenia: ASH guideline update. Hematology Am Soc Hematol Educ Program.

[97] Provan D, Stasi R, Newland AC, Blanchette VS, Bolton-Maggs P, Bussel JB, Chong BH, Cines DB, Gernsheimer TB, Godeau B, Grainger J, Greer I, Hunt BJ, Imbach PA, Lyons G, McMillan R, Rodeghiero F, Sanz MA, Tarantino M, Watson S, Young J, Kuter DJ (2010). International consensus report on the investigation and management of primary immune thrombocytopenia. Blood..

[98] Ghanima W, Godeau B, Cines DB, Bussel JB (2012). How I treat immune thrombocytopenia: the choice between splenectomy or a medical therapy as a second-line treatment. Blood..

[99] Ramachandran S, Zaidi F, Aggarwal A, Gera R (2017). Recent advances in diagnostic and therapeutic guidelines for primary and secondary hemophagocytic lymphohistiocytosis. Blood Cells Mol Dis.

[100] Henter JI, Horne A, Aricó M, Egeler RM, Filipovich AH, Imashuku S, Ladisch S, McClain K, Webb D, Winiarski J, Janka G (2007). HLH-2004: diagnostic and therapeutic guidelines for hemophagocytic lymphohistiocytosis. Pediatr Blood Cancer.

[101] Kleynberg RL, Schiller GJ (2012). Secondary hemophagocytic lymphohistiocytosis in adults: an update on diagnosis and therapy. Clin Adv Hematol Oncol.

[102] Chalmers E (2011). Purpura fulminans: recognition, diagnosis and management. Arch Dis Child.

[103] Colling ME, Bendapudi PK (2018). Purpura fulminans: mechanism and management of dysregulated hemostasis. Transfus Med Rev.

[104] Bendapudi PK, Robbins A, LeBoeuf N, Pozdnyakova O, Bhatt A, Duke F, Sells R, McQuiston J, Humrighouse B, Rouaisnel B, Colling M, Stephenson KE, Saavedra A, Losman JA (2018). Persistence of endothelial thrombomodulin in a patient with infectious purpura fulminans treated with protein C concentrate. Blood Adv.

[105] Sakashita K, Murata K, Takamori M (2018). TAFRO syndrome: current perspectives. J Blood Med.

[106] Kawabata H, Takai K, Kojima M, Nakamura N, Aoki S, Nakamura S, Kinoshita T, Masaki Y (2013). Castleman-Kojima disease (TAFRO syndrome): a novel systemic inflammatory disease characterized by a constellation of symptoms, namely, thrombocytopenia, ascites (anasarca), microcytic anemia, myelofibrosis, renal dysfunction, and organomegaly : a status report and summary of Fukushima (6 June, 2012) and Nagoya meetings (22 September, 2012). J Clin Exp Hematop.

[107] Semra P (2018). Tafro syndrome: critical review for clinicians and pathologists. Crit Rev Oncol Hematol.

[108] Louis C, Vijgen S, Samii K, Chalandon Y, Terriou L, Launay D, Fajgenbaum DC, Seebach JD, Muller YD (2017). TAFRO syndrome in Caucasians: a case report and review of the literature. Front Med.

[109] Guo CT, Lu QB, Ding SJ, Hu CY, Hu JG, Wo Y, Fan YD, Wang XJ, Qin SL, Cui N, Yang ZD, Zhang XA, Liu W, Cao WC (2016). Epidemiological and clinical characteristics of severe fever with thrombocytopeniasyndrome (SFTS) in China: an integrated data analysis. Epidemiol Infect.

[110] Oh WS, Yoo JR, Kwon KT, Kim HI, Lee SJ, Jun JB, Ryu SY, Kim HA, Hur J, Wi YM, Lim MH, Heo ST (2017). Effect of early plasma exchange on survival in patients with severe fever with thrombocytopenia syndrome: a multicenter study. Yonsei Med J.

[111] Afdhal NH, Giannini EG, Tayyab G, Mohsin A, Lee JW, Andriulli A, Jeffers L, McHutchison J, Chen PJ, Han KH, Campbell F, Hyde D, Brainsky A, Theodore D (2012). Eltrombopag before procedures in patients with cirrhosis and thrombocytopenia. N Engl J Med.

[112] Loudin M, Ahn J (2017). Portal vein thrombosis in cirrhosis. J Clin Gastroenterol.

